# Empowering knowledge dissemination: a 5A model for health professionals in IVF health communication through short videos

**DOI:** 10.3389/fpubh.2026.1793096

**Published:** 2026-05-28

**Authors:** Jiacheng Dan, Shuo Li, Lei He

**Affiliations:** West China Second University Hospital, Sichuan University, Chengdu, Sichuan, China

**Keywords:** health communication, health professionals, in-vitro fertilization (IVF), patient education, short videos

## Abstract

**Background:**

Patients increasingly rely on social media for IVF-related information; however, the quality of such content remains suboptimal. Health professionals who receive systematic medical education possess disciplinary knowledge that supports the production of accurate health communication. Engaging in health communication can strengthen their ability to translate specialized knowledge into accessible language, a skill that also connects with the competence of continuing medical education.

**Objectives:**

This study aimed to evaluate IVF-related short videos on Douyin and to propose a practical model, 5A, to help health professionals create effective health communication videos.

**Methods:**

A total of 137 IVF-related short videos were evaluated using the DISCERN instrument and a sharing engagement index. Multivariable linear regression and Beta regression models were used to identify factors associated with video quality and dissemination efficiency, respectively. Stratified analyses and Spearman’s correlations were further conducted to examine variable distributions and inter-index associations. Additionally, semi-structured interviews with eight stakeholders were conducted to identify production barriers. The findings were integrated to construct the 5A model, a practical instructional model for health education.

**Results:**

The mean DISCERN score was 43.30 (SD: 7.87). A multivariable analysis revealed that longer video duration [*β* = 2.70, 95% CI: (0.46, 4.94)] and no name tag [*β* = −3.60, 95% CI: (−6.17, −1.02)] were significantly associated with DISCERN scores. Beta regression models revealed that video duration [OR = 1.30, 95% CI: (1.07, 1.57)] and follower count [OR = 1.07, 95% CI: (1.03, 1.11)] were significantly associated with the sharing engagement index. Additionally, the sharing engagement index showed a weak positive correlation with DISCERN scores (*r_s_* = 0.2, *p* = 0.017). Qualitative analysis identified five themes: content quality, language expression, filming presentation, editing production, and speaker characteristics. Based on the results, the 5A model was proposed, consisting of accessibility of language, adaptation of setting, authorization of physicians, attractiveness of content, and appeal of editing.

**Conclusion:**

Although algorithms play a role in dissemination, high-quality professional content remains essential for effective dissemination. This study advocates for integrating communication skills into medical health, highlighting the fact that health communication is a teachable competency. The 5A Model offers an actionable framework that enables health professionals to address the training gap to be effective public educators.

## 1 Introduction

In recent years, infertility has become a major public health challenge worldwide (1)^.^ Due to complex factors, including intensifying life pressures (2) and delayed marriage and childbearing (3), the infertility rate among the reproductive-age population has shown a marked upward trend in China (4). Consequently, assisted reproductive technologies, represented by in vitro fertilization (IVF), are increasingly being applied in clinical practice, becoming a crucial clinical pathway for couples seeking to overcome their fertility barriers (5). However, the IVF journey is often characterized by lengthy cycles, substantial financial costs, and uncertain outcomes (6). A study showed that individuals undergoing assisted reproductive technologies commonly experience varying degrees of anxiety and depression (7, 8). These challenges frequently increase their needs for timely medical information, emotional support, and shared experiential knowledge. In the context of limited consultation time and insufficient continuous support within traditional healthcare settings, patients are increasingly turning to the internet and social media platforms to proactively seek accessible medical information and guidance (9, 10). Characterized by intuitiveness, fragmentation, and high interactivity, short videos have transcended traditional media to become a ubiquitous channel for information dissemination, such as Douyin and Kuaishou. According to the 56th Statistical Report on China’s Internet Development, released by the China Internet Network Information Center, as of June 2025, the short-video user base reached 1.068 billion, accounting for 95.1% of all internet users (11). Data from the 2024 Douyin Health Annual Report revealed that 75% of users actively seek health-related content, with 79% identifying short videos as their primary source of health information. Guided by the “Healthy China 2035” policy, the media have strengthened the communication of health science through new media for health education. Concurrently, an increasing number of health professionals have established a presence on short video platforms. Although such content generated by health professionals is typically perceived as more credible (12), significant heterogeneity in its quality persists. Existing research has indicated that challenges such as the insufficient translation of specialized medical jargon into accessible language (13), the excessive fragmentation of complex health information (14), a lack of humanistic care in narrative styles (15), and non-standardized video production techniques (16) may collectively hinder the effective dissemination of scientific knowledge.

This study aimed to identify the core factors influencing both information quality and dissemination effectiveness and to develop a practical, actionable health communication model for health professionals, empowering them to become more effective public health educators.

## 2 Materials and methods

### 2.1 Study design and research process

Quantitative analysis can only capture the explicit features and measurable quality scores of a video. Therefore, this study also adopted qualitative interviews to uncover potential factors. The research was conducted in four sequential phases, including video searching, quantitative assessment of short videos using validated evaluation tools, organizing qualitative interviews, and statistical analysis.

### 2.2 Short video search strategy

The data were obtained from Douyin on 30 September 2025. Searches were conducted using the following keywords: “试管婴儿,” “IVF,” “胚胎移植,” (embryo transfer), and “人工辅助移植,” (artificially assisted transplantation). To avoid personalized recommendations, searches were conducted while not logged in and ranked by the platform’s algorithmic, comprehensive sorting, with no publication date set. The first 60 short videos from each search query were selected based on prior research and user browsing habits. These videos can be viewed without registration or login. All selected videos are publicly available on Douyin, could be freely accessed on electronic devices without any restrictions, and are presented in Mandarin Chinese.

The exclusion criteria applied in this study include the following: (1) videos unrelated to IVF or lacking educational content, (2) duplicate content, (3) commercially produced videos, and (4) non-original content.

### 2.3 Characteristics of short videos

This study recorded the characteristic information of selected short videos, including video duration, name tag, shooting settings, dress code, and expression style (Supplementary Table S1).

### 2.4 DISCERN (quality evaluation of short videos)

This study selected the validated DISCERN assessment tool (17), consisting of 16 items: 8 reliability assessment items, 7 quality of information on treatment choices assessment items, and 1 overall quality assessment item, to evaluate short videos of health education content. The evaluation was independently conducted by two obstetricians who were experts in this field, using a 5-point Likert scale (1 - no, 2–4 - partially, and 5 - yes). The total scores ranged from 16 to 80. All disagreements were evaluated by a third senior expert. Cohen’s kappa coefficient was 0.800 (*p* < 0.01), indicating inter-rater reliability. Cronbach’s alpha of this scale was 0.866 in this study.

### 2.5 Sharing engagement of short videos index

To evaluate short-video engagement, this study constructed an engagement index based on the platform’s data characteristics and prior research, incorporating three key indicators: likes, comments, and shares. These three behaviors collectively represented users’ responses to video content, with likes reflecting immediate attitudes, comments indicating deeper communication, and shares representing dissemination willingness. To quantify secondary dissemination behavior while accounting for the high variance across disparate scales of video traffic, total engagement counts were transformed into the Sharing Engagement Index via the saturation curve illustrated in Supplementary Figure S1. This index, which captures the relative contribution of sharing to total user engagement, is calculated as follows:

$$\begin{array}{l} \text{Sharing Engagement of Short Videos Index}=\\ (\frac{\text{Numbers of Shares}}{\text{Number of Likes}+\text{Comments}+\text{Shares}})\times 100\% \end{array}$$

To control for differences in posting frequency, this study calculated a normalized indicator: daily average interactions-total engagement/days since upload.

### 2.6 Qualitative research

A descriptive qualitative research approach was adopted. Participants were recruited through purposive sampling conducted by the researcher, JC. The inclusion criteria required participants to have at least 5 years of professional experience in their respective fields (medical fields, media communication education, and mainstream broadcasting stations) and to be actively involved in, or previously engaged in, work related to health communication. All interviews were conducted by JC, who possesses a background in communication studies and currently works in medical education at a hospital. This dual background facilitated a deep understanding of medical terminology while maintaining a professional perspective on communication strategies. Prior to the interview, the purpose and content of the study were explained. Verbal informed consent was obtained from all participants, including permission for audio recording and awareness of their right to withdraw at any time. One-to-one interviews were conducted in a quiet room. The interview guide (Supplementary data S1) was used, with questions flexibly adjusted based on individual circumstances. The entire interview was audio-recorded, and the average duration was 30 min.

### 2.7 Statistical analysis

**Figure 1:**
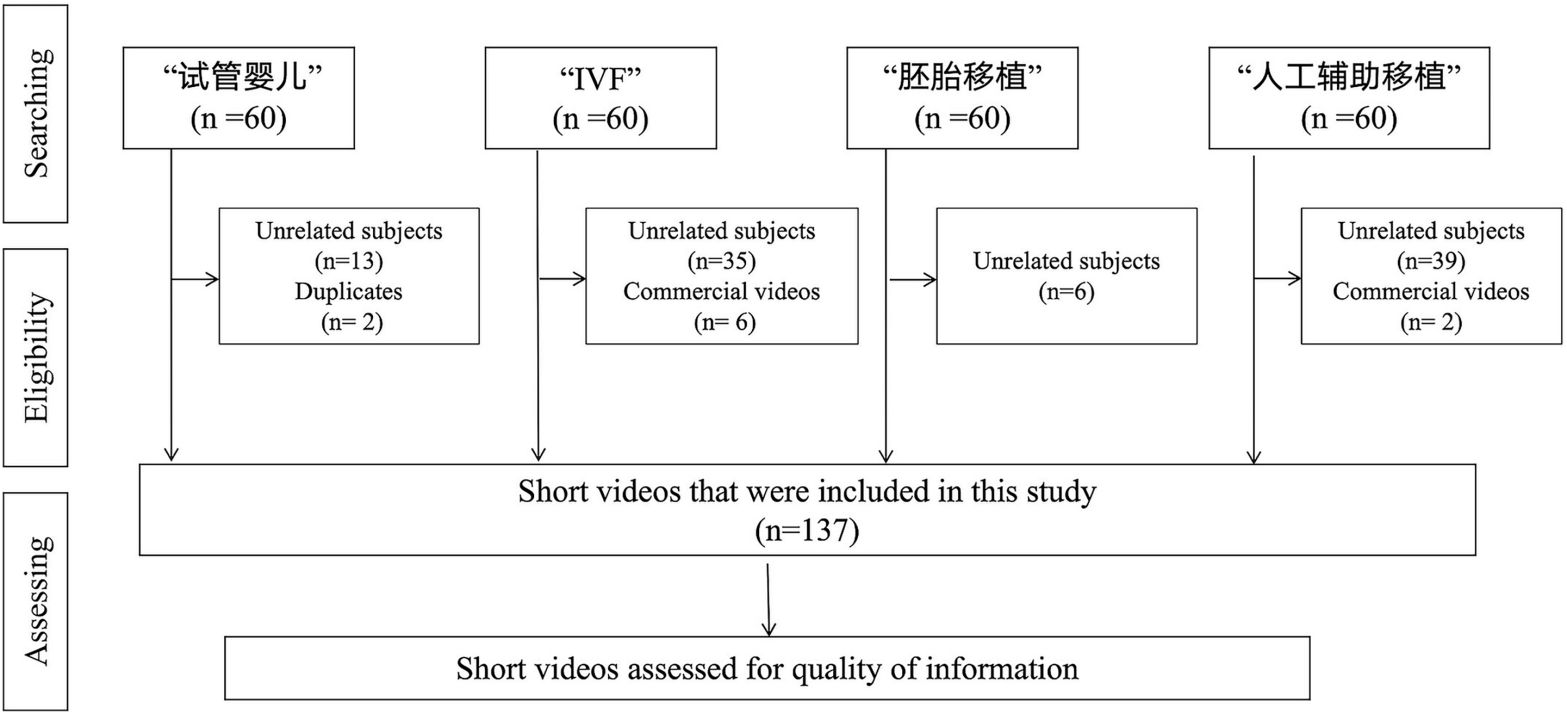
Short videos screening process.

A total of 240 short videos were retrieved through searching. After screening based on inclusion and exclusion criteria, 137 eligible short videos were selected for quality assessment, as shown in Figure 1. Video characteristics were described as medians (interquartile ranges, IQRs) for continuous variables because they were non-normally distributed, while categorical variables were reported as frequencies and percentages (%). Comparisons between groups were performed using the Mann–Whitney U-test for continuous data and Fisher’s exact test for categorical data. All statistical tests were two-sided, with a *p*-value of < 0.05 defining statistical significance.

Stratified analyses were performed to evaluate the distribution of DISCERN scores across different categorical video parameters. Differences between groups were assessed using the *t*-test, with results visualized using boxplots, where the central line represents the median and the box plot denotes the interquartile range.

Multivariable linear regression models were used to estimate regression coefficients (β) and 95% confidence intervals (CIs) for associations between video-related parameters and DISCERN scores. The regression models were adjusted for name tag, dress code, shooting environment, expression style, video duration, days since publication, followers count, and shares count. Multicollinearity was assessed using variance inflation factors (VIFs) to ensure the stability of the estimates and models.

Multivariable beta regression models were used to estimate odds ratios (ORs) and 95% confidence intervals (CIs) for associations between video-related parameters and the sharing engagement index. The regression models were adjusted for name tag, dress code, shooting environment, expression style, video duration, days since publication, and follower count. Since the index contained values of 0, a transformation *y*’ = [*y*(*n*-1) + 0.5]/*n* was applied to compress the data into the (0, 1) interval (18).

Spearman’s rank correlation analysis was performed to quantify the degree of association between DISCERN scores and the sharing engagement index. A scatter plot with a fitted regression line was used to visualize the association.

Non-normal distribution of continuous variables was log-transformed prior to inclusion in all of the above models. All analyses were performed using R 4.3.3 (2024-02-29). A two-sided *p-*value of *<* 0.05 was considered statistically significant.

For qualitative data, audio recordings were independently transcribed by two researchers (JC and LH) within 24 h of each interview to ensure data integrity. All interview data were analyzed using the Colaizzi 7-step method to ensure rigorous and systematic extraction of themes (19). This method allowed for the identification of core themes, including content quality, language expression, and speaker characteristics. Two researchers conducted independent coding, followed by multiple rounds of discussion to compare coding results. Any discrepancies were resolved through consensus. All data were handled with strict privacy protections: each participant was assigned a unique code during transcription (P1–P8) to ensure anonymity. Access to the audio files and transcripts is restricted to two researchers (JC and LH). Recruitment was concluded when the analysis of the final two participants (P7 and P8) yielded no new themes or sub-themes.

## 3 Results

A total of 240 short videos were downloaded through the search. Following the inclusion and exclusion criteria, 103 videos were excluded after individual review. Ultimately, this study evaluated the quality of 137 short videos, with the screening process illustrated in Figure 1.

### 3.1 Quantitative research findings

#### 3.1.1 Basic characteristics of the videos

Among the 137 videos included in the analysis, the median video duration was 58 s, and the median number of days since publication was 124 days (Table 1). Health-related dress and health-related shooting environments were prevalent across 92.7 and 81.0% of the videos, respectively. A one-way personal statement was the primary delivery style (75.9%), and professional name tags were incorporated in 46.7% of the total sample. When comparing the two quality groups, videos rated as “Good or above” (*n* = 24) were significantly longer than those rated “Fine or below” (*n* = 113; median 77.5 vs. 56 s, *p* = 0.017). Regarding user engagement metrics, although the number of likes, comments, and daily average interactions did not show statistically significant differences, there was a marginal trend toward higher sharing behaviors for the higher-quality videos (*p* = 0.099). Furthermore, no significant differences were observed between the two quality strata in dress code, shooting environment, or the presence of a name tag (all *p* > 0.05).

**Table 1:** Characteristics of included short videos.

Characteristics	Total	Good or above	Fine or below	*P*
	(*n* = 137)	(*n* = 24)	(*n* = 113)	
Video duration, median(IQRs), seconds	58 (42, 92)	77.50 (48.75, 140.50)	56 (42, 79)	**0.017**
Days since publication, median(IQRs), days	124 (84, 232)	110 (78, 178.25)	125 (86, 234)	0.477
Engagement metrics, median(IQRs)				
Number of likes	205 (74, 881)	347 (94, 1863.75)	196 (73, 781)	0.262
Number of shares	60 (14, 441)	117 (30.25, 1181.50)	58 (14, 302)	**0.099**
Number of comments	10 (3, 66)	13.50 (3.75, 214.75)	9 (3, 52)	0.401
Number of followers	27,000 (3,929, 76,000)	21,000 (9610.50, 68,250)	31,000 (1714, 81,000)	0.874
Daily average interactions, median(IQRs)	2.73 (0.62, 11.32)	2.28 (0.38, 16.60)	3.08 (1.17, 9.14)	0.246
Dress code, *n* (%)				0.380
Health-related (White coat and Surgical gown)	127 (92.7)	21 (87.5)	106 (93.8)	
Others	10 (7.3)	3 (12.5)	7 (6.2)	
Expression style, *n* (%)				1.000
Questions and answers	33 (24.1)	6 (25.0)	27 (23.9)	
Personal statement	104 (75.9)	18 (75.0)	86 (76.1)	
Shooting environment, *n* (%)				0.164
Health-related	111 (81.0)	17 (70.8)	94 (83.2)	
Others	26 (19.0)	7 (29.2)	19 (16.8)	
Name tag, *n* (%)				0.262
Yes	64 (46.7)	14 (58.3)	50 (44.2)	
No	73 (53.3)	10 (41.7)	63 (55.8)	

#### 3.1.2 Associations between DISCERN score and video-related parameters

**Figure 2:**
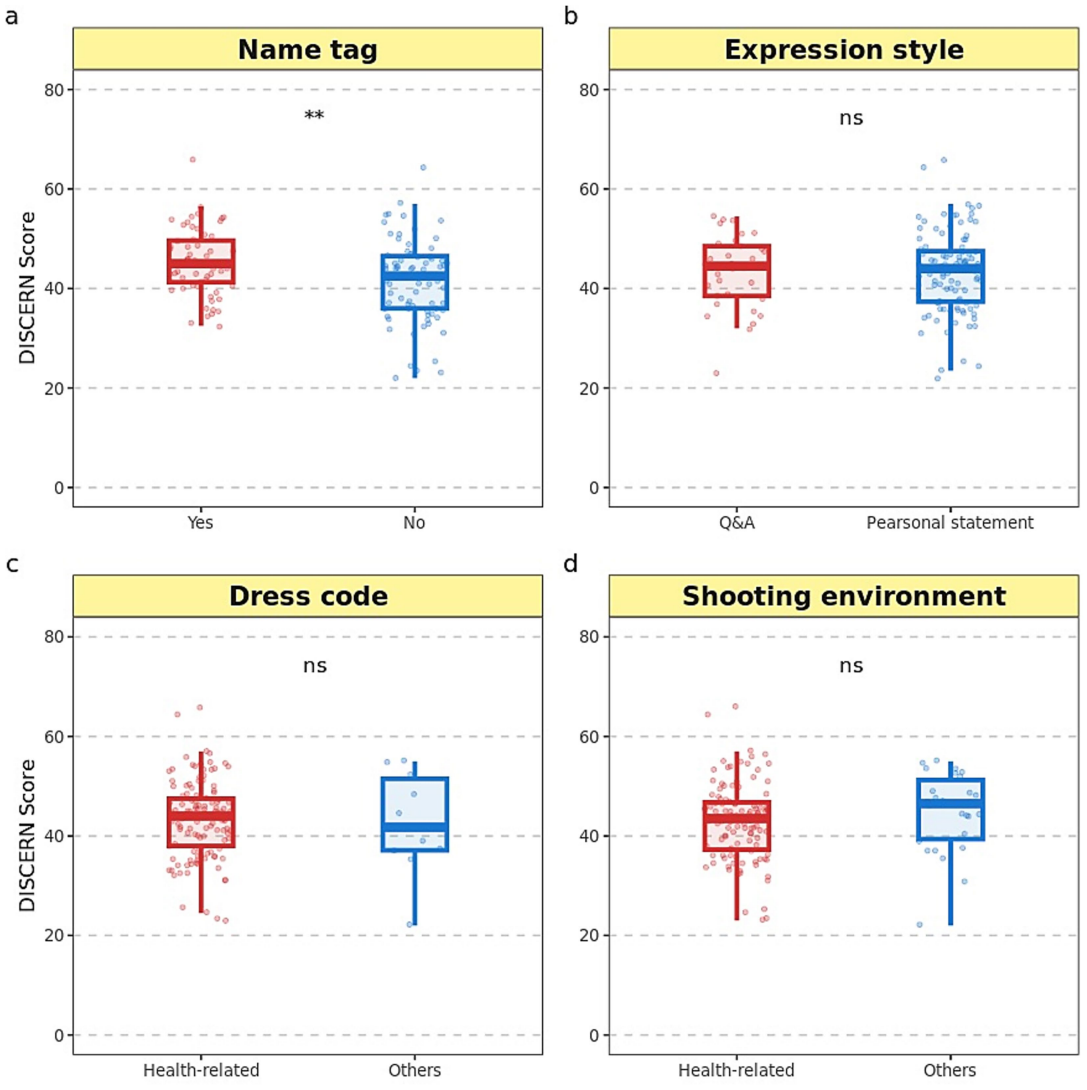
Distribution of DISCERN quality scores across different categorical features of IVF-related short videos. Boxplots display the informational quality of **(a)** Name tag, **(b)** Expression style, **(c)** Dress code, and **(d)** Shooting environment. Statistical differences between groups were assessed using the Mann–Whitney *U*-test (*p* < 0.01; ns: not significant). The boxes represent interquartile ranges (IQRs), the thick line denotes the median, and the whiskers extend to 1.5 times the IQRs. Individual data points are plotted as dots.

We first explored the distribution of DISCERN scores across different categorical features. The boxplot revealed that videos featuring a name tag had significantly higher median quality scores than those without (Figure 2a, *P* < 0.01), whereas no statistically significant differences were observed for expression style, dress code, or shooting environment (Figures 2b–d; all ns). To further quantify these associations while adjusting for other confounders, a multivariable linear regression model was constructed (Table 2). Specifically, video duration was significantly and positively associated with higher DISCERN scores (B = 2.70, *p* = 0.019), indicating that longer content tended to provide more comprehensive information. Furthermore, the absence of a professional name tag was a significant negative predictor, with scores averaging 3.60 points lower than those for videos featuring clear identity markers (*p* = 0.007). Conversely, shares count and other visual cues (e.g., dress code) did not exhibit statistically significant differences in this model (*p* > 0.05).

**Table 2:** Multivariable linear regression analysis between DISCERN score and video-related parameters.

Variables	B	BSE	β	βSE	B 95% CI	β 95% CI	Statistic	*P*
Duration (seconds)	2.70	1.13	0.21	0.09	(0.46, 4.94)	(0.04, 0.38)	2.38	**0.019**
Days since publication	0.36	0.79	0.04	0.09	(−1.20, 1.93)	(−0.13, 0.21)	0.46	0.645
Number of followers	−0.40	0.21	−0.17	0.09	(−0.81, 0.02)	(−0.34, 0.01)	−1.90	0.060
Shares	0.39	0.32	0.12	0.09	(−0.23, 1.01)	(−0.07, 0.30)	1.24	0.218
Name tag								
Yes	ref							
No	−3.60	1.30	−0.46	0.17	(−6.17, −1.02)	(−0.78, −0.13)	−2.77	**0.007**
Dress code								
Health-related	ref							
Others	−4.19	2.93	−0.53	0.37	(−9.98, 1.61)	(−1.27, 0.20)	−1.43	0.155
Shooting environment								
Health-related	ref							
Others	2.19	1.96	0.28	0.25	(−1.68, 6.05)	(−0.21, 0.77)	1.12	0.266
Expression style								
Questions and answers	ref							
Personal statement	−0.17	1.55	−0.02	0.20	(−3.24, 2.90)	(−0.41, 0.37)	−0.11	0.912

B: unstandardized regression coefficient; B.SE: standard error of the unstandardized coefficient; β: standardized regression coefficient; β.SE: standard error of the standardized coefficient; CI: confidence interval; Statistic: t-statistic; P: *p*-value. Duration (seconds), days since publication, and number of followers, shares were log-transformed. Model fit: R^2^ = 0.150, adjusted R^2^ = 0.096, F(8,128) = 2.813, *p* = 0.007.

#### 3.1.3 Associations between sharing engagement index and video-related parameters

**Figure 3:**
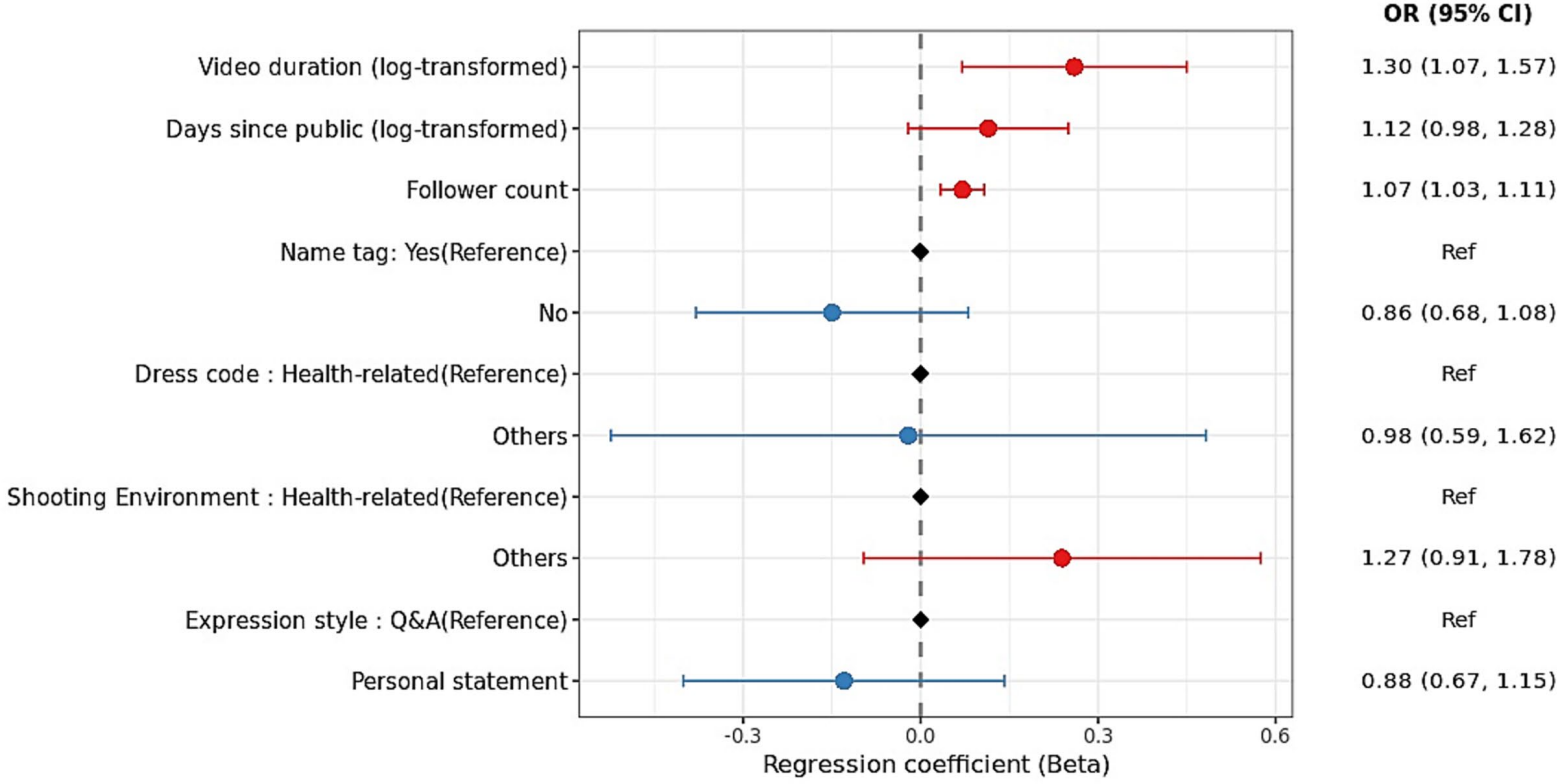
Forest plot of factors associated with the sharing engagement index based on beta regression analysis. The forest plot displays the multivariable associations with the sharing engagement index. Red points represent positive associations (*β* > 0), while blue points denote negative associations (*β* < 0). Black diamonds represent the reference categories (“Ref”). Values on the right indicate the exponentiated coefficients (OR) with their corresponding 95% confidence intervals. Video duration and days since publication were log-transformed.

Beta regression models were used to identify key determinants of the sharing engagement index, as visualized in the forest plot (Figure 3). After adjusting for confounding variables, video duration showed a significant positive correlation with the sharing engagement index [OR = 1.30, 95% CI: (1.07, 1.57)]. Similarly, follower count was positively associated with higher engagement scores [OR = 1.07, 95% CI: (1.03, 1.11)], reflecting both the cumulative nature of video reach. By comparison, categorical features, including professional name tags, dress code, shooting environment, and expression style, exhibited varied directional trends but did not reach statistical significance (all *p* > 0.05). For instance, although videos without professional name tags showed a lower propensity for being shared (OR = 0.86, 95% CI: 0.68–1.08), this association did not reach statistical significance. These findings suggest that information volume and the duration of platform exposure are more consistent correlates of sharing behavior than the examined visual and delivery attributes.

#### 3.1.4 Associations between DISCERN score and sharing engagement index

**Figure 4:**
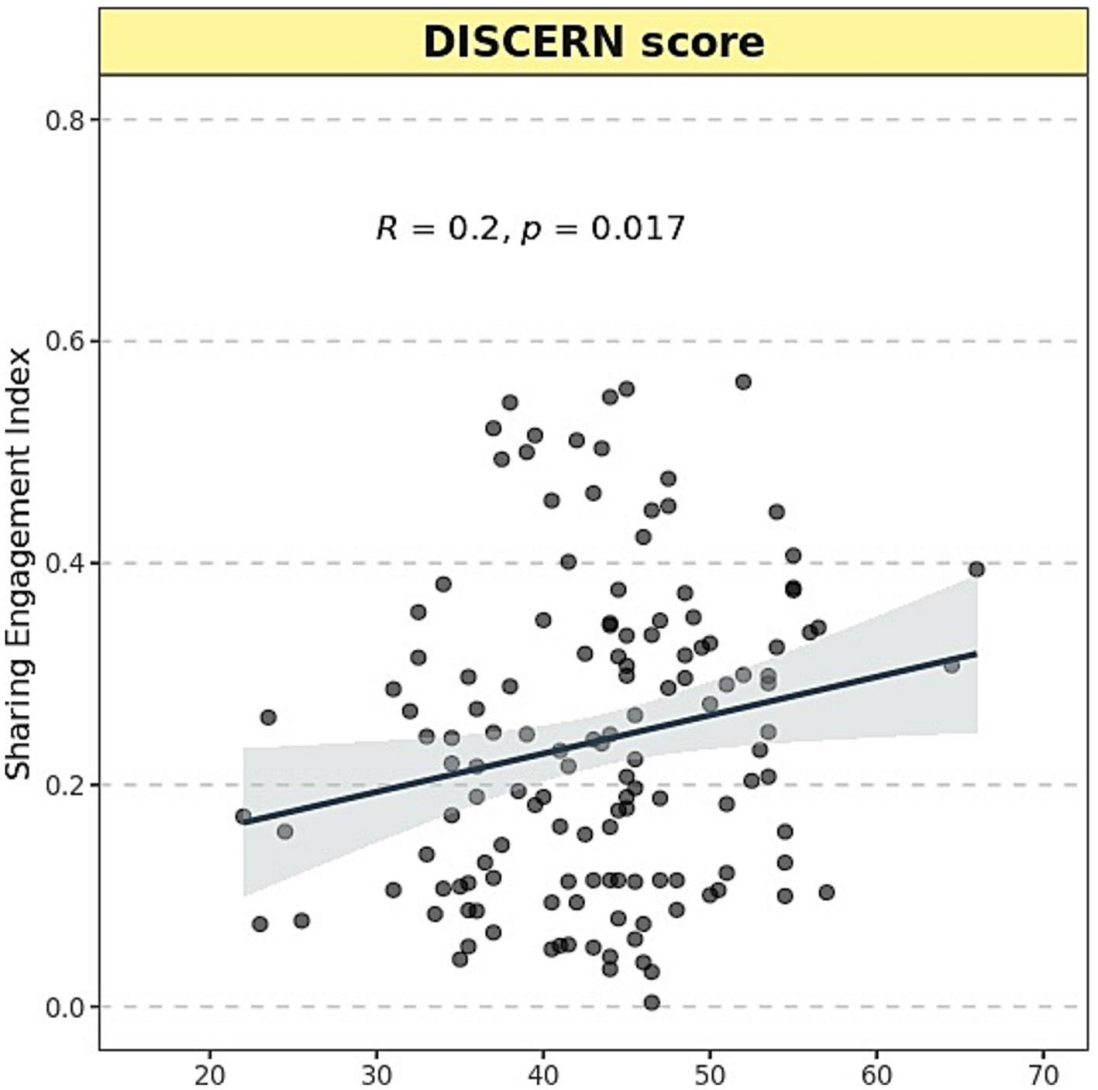
Correlation between DISCERN scores and the sharing engagement index. The scatter plot illustrates the relationship between the informational quality of videos (DISCERN score) and their dissemination level (sharing engagement index). The solid line represents the fitted linear regression, with the shaded area indicating the 95% confidence interval. Spearman correlation coefficient (*R*) and the corresponding *p*-value are provided.

Finally, we evaluated the relationship between the two primary characteristics assigned to the short videos: informational quality (DISCERN score) and user engagement (sharing engagement index). As illustrated in the scatter plot (Figure 4), a Spearman’s rank correlation analysis revealed a significant positive association between content quality and sharing behaviors (*R* = 0.2, *p* = 0.017). The fitted regression line indicates that, as DISCERN scores increased, the sharing engagement index increased accordingly. These results demonstrate a statistically significant, albeit weak, linear association between the content quality scores and the calculated dissemination metrics in the sampled IVF-related short videos.

### 3.2 Qualitative research findings

A purposive sampling method was used to recruit a multidisciplinary panel of 8 interviewees (*n* = 8), covering three core fields: medical fields, media communication education, and mainstream broadcasting stations. All participants were coded as P1 to P8, with their gender, professional designation, and institutional affiliation specified. Specifically, a male attending physician (P1) and a female attending physician (P2) are both from a grade A tertiary hospital. A male senior nurse (P3) and a female senior nurse (P4) are both affiliated with a grade A tertiary hospital. P5 is a female lecturer formally affiliated with a regular institution of higher education related to media and communication. A male associate professor (P6) is from the university with media-related disciplines. Two female staff members from the provincial-level television station were included: one is the chief director, coded as P7, and the other is the director, coded as P8. Data saturation was achieved with this sample size, as no new themes emerged during the final interviews.

Qualitative research identified five topics, including content quality, language expression, filming and presentation, editing and production, and speaker characteristics. Table 3 presents a comprehensive summary of these themes along with their respective subthemes and illustrative quotes.

**Table 3:** Summary of qualitative themes, subthemes, and illustrative quotes.

Themes	Subthemes	Illustrative quotes	ID
Content Quality	Professional Depth	“The content is highly focused and not superficial.”	P6
		“…You should know your area deeply and focus on the latest findings that are supported by evidence.”	P1
		“It is highly professional, with relevant data clearly presented.”	P5
	Viewers’ Perspective	“People can understand this video easily and even people with no background in the medical industry can understand its meaning.”	P2
		“The topic is clear with its perspective of patients to make it be simple, clear, and excellent.”	P7
		“Storytelling is actually a very effective approach.”	P8
		“They used the hot topics that was how many injections were needed for in vitro fertilization.”	P3
Language Expression	Accessible Expression	“I think the improved comprehensibility mainly comes from its colloquial expression, which does not compromise on professionalism at the same time.”	P6
	Expression Style	“Actually, their articulation is quite clear, and their explanations are easy to follow.”	P8
		“…The format is question-and-answer, instead of a one-way lecture-style delivery.”	P1
Filming and Presentation	Shooting Environment	“The hospital environments, the medical uniforms, and the speaker’s mature age all contribute to a high level of credibility.”	P2
		“A doctor sitting in their own consulting room comes across as very professional.”	P7
	Shooting Quality	“… Some clips were shot with mobile phones, and the image quality was not particularly good, which directly impairs the viewer’s viewing experience.”	P8
Editing and Production	Importance of Subtitle	“… Key points were presented with subtitles, and some core terms were bold to serve as a reminder for the viewer.”	P2
		“It also included supporting visuals and the key text was highlighted in red for emphasis.”	P5
	Credibility Labeling	“Perhaps they are willing to use their hospital name because it carries the title of a public hospital.”	P1
	Short Video Duration	“… The answers were extremely concise and the video wrapped up in less than 30 s.”	P4
Speaker Characteristics	Appearance of Doctors	“His clothes and appearance are typical of a traditional doctor, making it easy for people to trust him.”	P3
		“This doctor is very experienced when I saw this appearance.”	P4
	Higher Credibility	“… You can emphasize the hospital, the doctor’s identity and qualifications, in order to enhance credibility through this kind of presentation.”	P8
		“This doctor speaks at a moderate pace and has a very professional image.”	P7

#### 3.2.1 Content quality

Health communication short videos should be both professional and interesting, enabling more viewers to understand the relevant information. It features a clear theme and adopts a patient-oriented perspective, presenting knowledge in a concise, straightforward, and well-polished manner. The video is accessible even to audiences with no medical background.

Content should be presented in a storytelling format, incorporating current hot topics and cases and using data to help the viewer understand the information easily. High-quality videos should be professional, evidence-based, and precisely focused on one’s own field, supported by the latest research. The way to boost understandability is to combine theories with ample specific cases and examples, adopt vivid storytelling, and select trending public concerns as topics.

#### 3.2.2 Language expression

In health communication videos, spoken language should be colloquial, and scientific knowledge should be conveyed clearly and straightforwardly. Interviewees emphasized maintaining a moderate speaking pace, appropriate pauses and intonation, clear articulation, and concise, to-the-point explanations. The content uses colloquial expressions without compromising professionalism and uses a natural question-and-answer format instead of rigid lecturing. Furthermore, their unscripted language is highly persuasive and easy for audiences to follow and understand.

#### 3.2.3 Filming and presentation

Shooting can take place in a real hospital environment to enhance the appeal of the video. Meanwhile, attention should be paid to visual clarity and lighting, enabling the viewer to gain a more intuitive understanding.

Professional settings—such as hospital environments, medical uniforms, and consulting rooms—greatly boost credibility. Thoughtful openings with real embryo footage and striking IVF visuals create a strong impact. However, poor-quality mobile phone footage negatively affects the viewing experience.

#### 3.2.4 Editing and production

In production editing, subtitles can be added to highlight key content, and explanations can be paired with images or animations to remove barriers to professional understanding. Additionally, adding a name tag can quickly enhance credibility and professionalism. The interviewee noted that their videos highlighted key text and combined live footage with animation. In addition, disclaimers and the names of public hospitals were included to ensure standardization and credibility.

#### 3.2.5 Speaker characteristics

When shooting health communication videos, wearing a white coat can enhance the viewers’ trust. The professional image of doctors can also boost credibility. Mature and experienced health professionals with professional appearances, appropriate speaking pace, and medical uniforms build strong credibility. Highlighting their identities and qualifications can further boost audience trust.

## 4 Discussion

This study assessed the quality of IVF short videos on Douyin and found that the overall quality of IVF-related short videos was suboptimal. Specifically, 81.8% of the analyzed content were rated as “Fair” or below according to the DISCERN instrument, consistent with findings from studies evaluating stroke nutrition (20) and autoimmune liver disease (21) content on the same platform. The prevalence of suboptimal content may compel clinicians to expend additional effort during outpatient consultations to correct misinformation encountered online, which not only increases their cognitive load but also risks eroding the foundation of trust. However, the underlying reasons for this phenomenon are multifaceted. First, it may be related to Douyin’s algorithm-driven traffic mechanism, which encourages creators to produce content that prioritizes entertainment over professional and rigorous health communication. The review mechanisms and industry standards for short-video platforms focused on healthcare and wellness may still be in the process of refinement. Additionally, health professionals often lack systematic training in communication studies when producing short-form health science videos, making it challenging for them to translate knowledge into visual content. Meanwhile, their focus remains on clinical work (22), leaving them with limited time to engage in health science communication.

To further examine factors influencing short videos quality, this study found significant differences in quality scores according to video duration and name tags. This may be attributable to the complexity of health communication, which involves specialized medical terminology, complex procedures, and significant patient variability (23), requiring creators to strike a balance between accuracy and comprehensibility. Longer video durations provide creators greater capacity to convey information, enabling thorough and rational explanations of complex medical topics. This enhanced the comprehensiveness and professionalism of the content. Short videos with a name tag (name and hospital information) achieve significantly higher quality scores. Name tags, as a form of identity verification, enhance the transparency and credibility of short-video content through their real-name authentication feature. Existing research indicated that greater transparency of sources in health communication content better facilitated viewers’ assessment of its scientific validity and reliability (24). Additionally, short videos with name tags may have a more explicit educational purpose, and their creation process may involve content review and technical support from departmental or hospital teams, thereby enhancing video quality. This study also found that the sharing engagement index of short videos exhibited a significant positive correlation with video quality scores. While “likes” and “comments” may represent transient emotional reactions or general engagement, sharing behavior entails a higher cognitive threshold and a social reputation cost (25). Additionally, users are more likely to disseminate content that they perceive as highly credible and beneficial to their social circles.

Given the gaps between clinical expertise and effective dissemination, this study integrated quantitative and qualitative findings to develop the 5A model, which refers to accessibility of language expression, adaptation of setting, authorization of physicians, attractiveness of contents, and appeal of editing. This model is designed as a pragmatic and actionable toolkit for health professionals to streamline the transformation of complex IVF knowledge into high-quality, authoritative, and engaging short-form video content.

By applying the 5A model, health professionals can create health communication content that is more professional and accessible, while aligning it with patients’ needs and the dissemination characteristics of various platforms. This may help address misinformation at the source by enhancing the visibility and credibility of professional content within the online information environment.

*Accessibility of language expression.* The key to enhancing the effectiveness of health communication is ensuring that medical information is linguistically accessible. Health professionals must maintain medical rigor while breaking down complex concepts using accessible language, balancing scientific accuracy with communicative clarity to achieve the goal of knowledge dissemination. The field of IVF is characterized by technical jargon, and when creators directly presenting it, it will raise the barrier to understanding for viewers. Rather than relying on dense medical terminology, information should be reconstructed using plain language and colloquial analogies. Health professionals may make obscure assisted reproductive technology knowledge accessible, thereby fulfilling the educational mission of health communication videos.

*Adaptation of setting.* Compared to written or oral health education communication, settings that reflect the features of treatment techniques can help viewers concretely visualize and understand complex medical processes. Considering the balance between technical rigor and accessibility, creators can scientifically plan filming locations by integrating the clinical characteristics of IVF treatment with content objectives. For instance, physicians can explain IVF classification and potential risks in consultation rooms, demonstrating medical rigor and ethics and introducing the embryo culture process in laboratories. By matching settings with health communication content, abstract medical knowledge can be transformed into clear, credible, and process-oriented technical pathways, which enables viewers to build a clear understanding and confidence in treatment while comprehending the science.

*Authorization of physicians.* Health professionals who have received systematic medical education possess disciplinary knowledge, which supports the production of accurate science communication. The white coat, as an iconic symbol of the medical profession, is the core visual representation of a doctor’s identity and directly influences perceptions of authority (26). Name tags are a crucial element for information annotation, providing viewers with direct access to doctors’ professional credentials (27). Therefore, health professionals should be encouraged to display these elements in short videos, as they represent the most direct way to build professional trust. Appearing on camera with their real names serves both as professional certification and as a way to build their personal brand. Name tags should be fixed in positions such as the bottom left or top right corner of the frame, where they are unlikely to obscure the main subject, including three essential elements: “Name + Affiliated Institution + Core Professional Title or the field of expertise”.

*Attractiveness of content.* In the information-saturated digital media environment, user attention is a scarce resource (28). For health communication short videos, accessibility is a prerequisite for professionalism to be valuable. Especially in the anxiety-ridden field of IVF, simply presenting “success rates for older women” or “treatment standards” in a straightforward manner is highly likely to be dismissed by users due to its dryness or because it evokes fears. Therefore, creators should adopt a viewer-centered approach, incorporating strategies such as narrative medicine to build emotional resonance through real case studies, thereby establishing a trust connection within seconds. This approach ensures medical accuracy while enhancing dissemination power and emotional resonance.

*Appeal of editing*. Video editing is not only the integration and splicing of footage, but rather a bridge connecting content and viewers. Excellent editing can precisely convey medical knowledge, enhance content appeal, and improve the accessibility of professional medical content through rhythm control, visual enhancement, and effective information delivery. For example, when explaining the differences in three generations of IVF technology, editing can construct a progressive logic of “definition explanation, applicable patient cases, and technical animation,” supplemented with keyword subtitles to visualize the principles. Furthermore, the essence of editing lies in controlling the rhythm of attention. Editing should use a rhythmic structure, such as a fast-paced opening, a moderated pace during key points, and a concise conclusion, to maintain engagement from viewers. In the “golden 3 seconds” opening, the editing rhythm needs to be fast-paced and impactful, utilizing a “question-based” strategy to quickly engage the viewer. For example, directly posing a question such as, “Can IVF be successful on the first try?” instantly captures viewers’ attention. When explaining complex processes such as “embryo culture,” creators can slow down the editing pace and, with some specific close-up shots, such as microscopic footage of embryos, in order to allow the viewer to clearly observe details, and the conclusion segment can recap core knowledge points in the form of quick cuts. This balanced approach ensures a high completion rate while also giving viewers the cognitive space to process complex information.

This study has several limitations that should be acknowledged. First, the data were retrieved from a single Chinese short-video platform. Nevertheless, the challenges posed by algorithm-driven content and misinformation have also been reported on other platforms. The framework of the 5A model may therefore have a certain degree of applicability across different regions and social media environments. The sampling strategy, which selected the first 60 videos based on the platform’s comprehensive sorting, introduced keyword and algorithmic bias. Second, the data represents a cross-sectional snapshot of dynamic metrics taken on a specific date. This does not account for the temporal volatility of engagement numbers, which fluctuates over time. Furthermore, these engagement metrics may be confounded by potential bot or paid-traffic behaviors, which could skew the perceived popularity of certain videos. Third, the raters of video quality were all from the same Grade A tertiary hospital. The raters may share similar educational backgrounds, and professional training may lead to a certain degree of framing bias. However, this hospital has strong academic influence and professional authority in reproductive medicine, ensuring a certain degree of professionalism and reliability in the rating results. Finally, despite reliability testing, the subjectivity of the DISCERN rating process cannot be completely eliminated.

## 5 Conclusion

To our knowledge, this is the first study to examine the production of IVF health communication short videos by health professionals. Our findings reveal that, despite the influence of algorithms, high-quality professional content remains essential. The 5A model equips health professionals with practical tools to improve dissemination. Consequently, we advocate for integrating communication skills into health communication. The 5A Model proposed in this study offers a systematic solution to the current training gap. By breaking down the complex production process into manageable modules, this model provides comprehensive guidance for health professionals lacking media experience. Meanwhile, this study encourages health professionals to participate in health communication, enabling them to become effective public educators who can bridge the gap between professional medical knowledge and patient understanding. To facilitate its broader implementation and long-term impact, we call for integrating this model into medical education curricula, thereby equipping future healthcare professionals with essential competencies in digital health communication.

## Data Availability

The raw data supporting the conclusions of this article will be made available by the authors, without undue reservation.
