# Supplementary material for: Empowering knowledge dissemination: a 5A model for health professionals in IVF health communication through short videos
Source: Front Public Health. 2026 May 28;14:1793096. doi: 10.3389/fpubh.2026.1793096 (PMC13253950; doi:10.3389/fpubh.2026.1793096)
Supplement: Supplementary file 1 [file Data_Sheet_1.docx]

# Table S1:Coding Scheme and Thematic Categories

| **Variable Name** | **Definition & Coding Categories** | |
| --- | --- | --- |
| Video Duration  (seconds) | The duration of each video in seconds. | |
| Days since publication | The number of days from the video’s release to the date of data collection. | |
| Dress Code | Doctors’ clothing characteristics featured in the videos | **Health-related (White coat and Surgical gown):** Creators wearing professional healthcare uniforms, specifically white coats or surgical gowns |
|  |  | **Others:** Any non-medical clothing, including casual wear, business suits |
| Expression Style | the way creators present content in the video | **Questions & Answers:** The video follows a question-and-answer structure, with questions from the audience or simulated common queries answered directly by the creator |
|  |  | **Personal statement:** Creators deliver one-way explanatory content or knowledge sharing by directly addressing the camera |
| Shooting Environment | Environmental and background characteristics of the video recording | **Health-related:** Associated with clinical or healthcare settings, such as hospitals, clinics, and medical consulting rooms |
|  |  | **Others:** All non-medical environments and backgrounds, such as home environments and outdoors |
| Name tag | Whether the video contains captions showing the individual’s full name, professional title, administrative position and institutional affiliation | |
| Commercially produced videos | Advertising video content including profit-making, brand promotion, product marketing, commercial publicity, rather than non-profit videos. An example is that the IVF promotional videos, which are produced by a professional team, convey professional medical strength and attract couples with fertility needs, fully conforming to the definition of commercially produced videos. | |
| Non-original content | video content that is not shot independently by the producer itself. Instead, it is composed by collecting and combining video clips downloaded from the Internet, without independent shooting and creation of the core audio-visual materials. An example of a non-original IVF video is an IVF video made by downloading online clips of IVF medical equipment, basic knowledge explanations and hospital scenes, then editing and combining these downloaded fragments without any independent shooting. | |

Table S2. Collinearity diagnostics (Variance Inflation Factors) for the multivariable linear regression model and beta regression model

| **Model** | **Variable** | **VIF** |
| --- | --- | --- |
| linear regression model | log_duration | 1.17 |
| linear regression model | log_days | 1.15 |
| linear regression model | log_followers | 1.18 |
| linear regression model | log_shares | 1.33 |
| linear regression model | name tag | 1.03 |
| linear regression model | dress code | 1.42 |
| linear regression model | Shooting Environmen | 1.44 |
| linear regression model | Expression style | 1.08 |
| beta regression model | log_duration | 1.09 |
| beta regression model | log_days | 1.11 |
| beta regression model | log_followers | 1.05 |
| beta regression model | name tag | 1.03 |
| beta regression model | dress code | 1.41 |
| beta regression model | Shooting Environmen | 1.44 |
| beta regression model | Expression style | 1.06 |

Data S1: Semi-Structured Interview Guide

**Invitation Paragraph**

I would like to invite you to participate in this interview. Before you decide whether you want to take part, it is important for you to understand the following information. Ask me if there is anything that is not clear or if you would like more information.

**What is the purpose of the study?**

The study aims to explore the creation, communication and quality of health communication short videos. A purposive sampling method was used to recruit interviewees from three fields: medical fields, media communication education and mainstream broadcasting station.

**Why have I been invited to take part?**

The inclusion criteria required participants had at least five years of professional experience in their respective fields and were actively involved in or had previously engaged in work related to health communication.

**What will happen if I take part?**

The study will ask you questions about your thoughts and opinion of following questions: 1) Do you usually watch health-related short videos? 2) What is your overall impression of health professionals creating science popularization videos? What characteristics do you think their short videos possess? 3) How can health professionals create high-quality science communication videos? 4) What factors enhance viewers trust and comprehension? 5) What methods can increase viewership or engagement? 6) What features from existing high-quality videos can be adopted? (By watching top-rated short videos) One-to-one interviews will be conducted in a quiet room. The entire interview will be audio-recorded and the duration of each interview will be about 30 minutes.

**Do I have to take part?**

Participation is completely voluntary, and declining to take part will not disadvantage you in any way. If you choose to take part, verbal informed consent was obtained from you, including permission for audio recording and awareness of your right to withdraw at any time. You are free to withdraw at any point during completion of the interview, without having to give a reason. Withdrawing from the study will not affect you in any way. Once you finish the interview, it will no longer be possible to withdraw from the study because the data will be fully anonymous. Please do not include any personal identifiable information in your responses.

**Data handling and confidentiality**

This research is anonymous. This means that other people will not be aware of your identity, and they will not be able to connect you to the answers you provide, even indirectly. According to Article 32 of the Measures for Ethical Review of Life Science and Medical Research Involving Human Subjects (issued by National Health Commission), research involving the use of human information or data that does not cause harm to participants, does not involve sensitive personal information, and has no commercial interests may be exempted from formal ethical review.

**What will happen to the results of the study?**

The results of the study will be summarised in this study and it will not be shared with any third parties.

Figure S1. Theoretical sensitivity analysis of the Sharing Engagement Index.


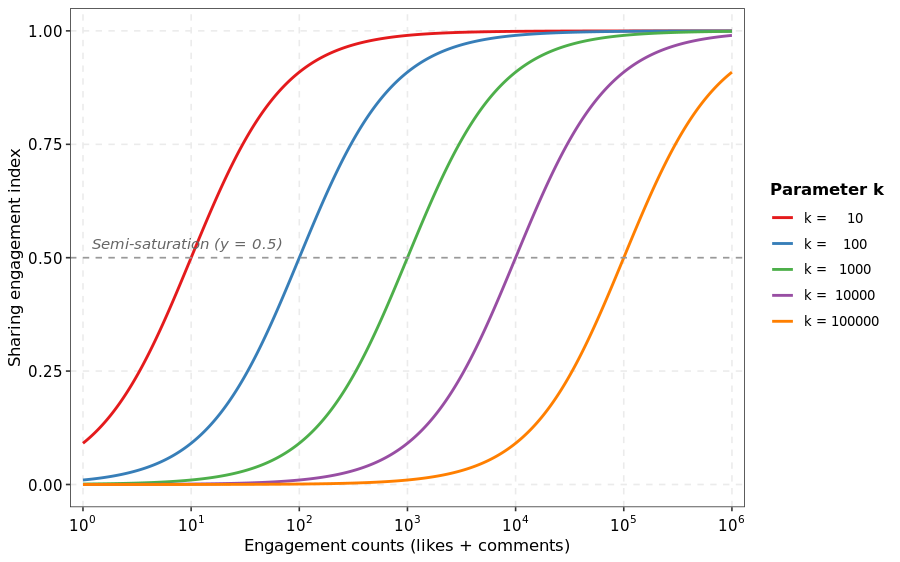


Figure S1. Theoretical sensitivity analysis of the Sharing Engagement Index. The curves illustrate the saturation-based normalization approach across six orders of magnitude in total engagement counts (10^0^ to 10^6^). The parameter k represents the semi-saturation constant, where the index value equals 0.5, demonstrating the metric's capacity to maintain comparability across disparate scales of video traffic.
